# Remote ischemic conditioning for safe cardioprotection in cardio-oncology?

**DOI:** 10.1007/s00395-026-01161-0

**Published:** 2026-02-05

**Authors:** Matthias Totzeck, Tienush Rassaf

**Affiliations:** Clinic for Cardiology and Vascular Medicine, West German Heart and Vascular Center, University Hospital Essen, Hufelandstr. 55, 45147 Essen, Germany

Anthracycline chemotherapy remains a cornerstone in the treatment of numerous solid tumors and hematological malignancies. It is well established that anthracyclines not only prolong cancer survival but also cause dose-dependent cardiotoxicity, including cancer therapy-related cardiovascular toxicity (CTR-CVT), ultimately leading to heart failure in cancer survivors [29, 33, 35]. Therefore, identifying new strategies to protect the heart while maintaining anticancer efficacy remains a central challenge in cardio-oncology. While conventional cardiac drugs (e.g., beta-blockers) or exercise-based programs have previously been tested in this context, current evidence should be interpreted with caution. Remote ischemic conditioning (RIC), induced by reversible episodes of ischemia/reperfusion in a distant organ (e.g., through repeated blood-pressure cuff inflations/deflations on a limb before, during, or after an acute event), has been empirically investigated for mitigating myocardial injury in the setting of reperfused acute myocardial infarction (repAMI) [11, 15]. RIC has emerged as a relatively simple, non-pharmacological intervention that is generally feasible in most emergency settings. Clinically, it appeared appealing due to its non-invasive nature and safety, requiring only short cycles of reversible limb ischemia/reperfusion via a standard blood pressure cuff [17, 32]. Although experimental models, including small and large animals, have largely produced promising outcomes, recent clinical trials in the context of repAMI have been underwhelming [20, 23]. Whether the beneficial effects of RIC can be extended to other cardiovascular diseases remains insufficiently explored.

Anabel Díaz-Guerra et al. from Borja Ibáñez’s laboratory present new data on the cardioprotective potential of RIC in reducing CTR-CVT in the current issue of *Basic Research in Cardiology* [3]. Their study demonstrates that RIC protects against anthracycline-induced cardiac injury without compromising antitumor efficacy in mice. Using a tumor-bearing mouse model—an ideal platform for cardio-oncology research [2]—the authors provide compelling evidence for RIC’s dual benefit. We commend the authors for their continued contributions to the field of cardioprotection, and this editorial will contextualize their findings within the broader scope of RIC research.

Ischemic conditioning, including remote forms such as RIC, was initially studied in the setting of repAMI. Among cardioprotective strategies tested in experimental models, ischemic conditioning remains one of the most effective, outperforming many approaches [14]. (R)IC interventions led to significantly reduced infarct sizes and preserved cardiac function [21]. The underlying protective mechanisms involve a complex signaling cascade with three critical components: (i) signal initiation in the distant organ, (ii) signal transmission via humoral or neural pathways, and (iii) activation of protective myocardial signaling during repAMI. The final myocardial response to RIC includes activation of receptors and cytosolic kinases that mitigate mitochondrial disintegration, calcium overload, and regulated cardiomyocyte death [17]. Additionally, RIC appears to influence the vasculature and endothelial function, which is essential for signal propagation [7, 8]. Our own work has highlighted the necessity of an intact endothelium for effective RIC signal generation [30]. Within the heart, RIC may also preserve coronary microcirculatory integrity and limit no-reflow injury during repAMI [13]. Initial randomized studies in ST-elevation myocardial infarction (STEMI) patients suggested that RIC, typically administered via limb cuff inflations before reperfusion, could reduce infarct size and biomarker release [1, 9]. These findings generated enthusiasm for RIC’s clinical application. However, subsequent large multicenter trials revealed a more complex picture [6, 10, 12]. Despite abundant preclinical evidence, many clinical trials failed to demonstrate meaningful benefits. To date, neither ischemic conditioning nor other cardioprotective therapies have become standard clinical practice for infarction or cardiac surgery, reflecting persistent translational challenges [14, 20]. Contributing factors include discrepancies between preclinical models and clinical populations—most animal studies use young, healthy rodents, whereas clinical patients are older and affected by comorbidities and concurrent medications that may alter RIC’s efficacy [4, 24]. Particularly in cardiac surgery, the effects of RIC have been inconclusive. While early-phase studies reported reductions in troponin levels, subsequent phase III trials showed no improvement in infarct size or clinical outcomes—likely due to confounding factors such as anesthetic regimens, hypothermia, and cardioplegic arrest [18, 27]. These limitations underscore that no conditioning strategy has yet been definitively proven to improve outcomes beyond timely reperfusion in myocardial infarction. Future cardioprotection trials must focus on appropriate patient selection and clinically meaningful endpoints. Patients with large infarct sizes or significant hemodynamic compromise may be more suitable candidates [19]. Additionally, endpoints should extend beyond acute injury metrics to include longer-term outcomes like heart failure [31].

The potential applications of RIC may, however, extend beyond repAMI. The chronic, cumulative cardiac damage from chemotherapeutic agents—particularly anthracyclines—represents another clinical target. Anthracyclines, though life-saving, can induce progressive cardiac injury through a plethora of mechanisms including oxidative stress, Top2β inhibition, and mitochondrial dysfunction [34]. Notably, these mechanisms overlap in part with those seen in repAMI [25, 34]. Thus, myocardial defense mechanisms mobilized during ischemia/reperfusion may also mitigate chemotherapy-induced damage [16, 28]. This hypothesis is supported by several preclinical studies. In rodents, RIC attenuated anthracycline-induced cardiomyocyte death and preserved mitochondrial function. A recent large-animal study in pigs further demonstrated that RIC administered prior to each doxorubicin dose preserved contractility and attenuated cardiac injury, confirming translational relevance [5]. However, early-phase clinical trials in cancer patients have yielded neutral outcomes. For example, in a pediatric cohort receiving anthracyclines, RIC failed to reduce troponin release or prevent cardiac dysfunction. Similarly, the adult ERIC-ONC trial found no differences in biomarkers or ejection fraction between RIC and control groups [26].

These findings raise important questions. Why did RIC prove successful in animals but not in clinical populations? One likely explanation lies in patient selection. Previous trials enrolled predominantly low-risk populations with mild expected cardiotoxicity, reducing the likelihood of detecting incremental benefit from RIC. This unfavorable signal-to-noise ratio may have obscured potential cardioprotection. Another key consideration is whether systemic protective signals might inadvertently shield tumor cells. Since both myocardium and tumors are metabolically active tissues, there is theoretical concern that pro-survival signaling could aid cancer cell survival. In one previous RIC-based trial, a higher number of cancer relapses and deaths occurred in the RIC arm. However, post-hoc analysis revealed baseline imbalances—RIC patients had more recurrent and metastatic disease—indicating that RIC was likely not responsible. Nonetheless, this observation emphasizes the need to monitor oncologic outcomes when testing cardioprotective strategies [22].

This is precisely why the study by Díaz-Guerra et al. is important. Using a tumor-bearing animal model, they evaluated both cardiac protection and tumor response concurrently. Their results are striking: limb RIC preserved cardiac function and reduced myocardial injury without impairing chemotherapy efficacy. Tumor size and apoptosis rates remained unchanged between RIC and control groups. This study provides the first proof-of-concept that RIC’s cardioprotection can occur without compromising oncologic treatment. The selectivity may derive from inherent differences between normal and malignant cells—cardiac cells benefit from moderate cytoprotective upregulation, whereas cancer cells, already harboring survival dysregulation, gain no added protection. These results may have timely clinical implications. The ongoing RESILIENCE trial, enrolling high-risk lymphoma patients and under the leadership of Dr. Ibanez, will test RIC’s cardioprotective effect prospectively. However, many open questions remain: What is the optimal frequency and duration of RIC in chemotherapy? Should RIC precede every infusion or be extended into the post-therapy remodeling phase? In animal studies, RIC was applied concurrently with each dose, a logical approach given the episodic cardiac injury. How does RIC perform in patients treated with other potential protective therapies (e.g., Sodium-Glucose Transport 2 inhibitors or statins) or exercise-based approaches? Future studies should finally also evaluate whether “booster” RIC cycles between treatments confer added benefit.

RIC’s favorable safety profile—limited primarily to cuff discomfort—offers a significant advantage over pharmacologic cardioprotection, which often cause off-target effects. Ongoing monitoring is warranted, but thus far, no safety issues have emerged across oncology trials. Broadly speaking, the application of RIC in cardio-oncology reflects the cautious optimism now guiding cardioprotection research. As the field matures, it becomes increasingly clear that successful translation depends on precise patient selection, sound trial methodology, and context-sensitive application (e.g., acute ischemia vs. chronic toxicity). The study under discussion pushes the envelope by extending RIC’s reach into chemotherapy-induced cardiomyopathy—an area well beyond its initial focus in infarction and surgery.

However, cautious interpretation remains warranted. Promising findings in controlled studies must ultimately withstand the complexities of clinical practice [28]. High-quality evidence will be essential before RIC can be formally recommended by guidelines. The current work contributes meaningfully to this evidence base, addressing a critical knowledge gap. The evolution of RIC exemplifies translational medicine: it bridges disciplines, challenges assumptions, and pursues innovation at the interface of cardiology and oncology. Its story continues to unfold—one chapter at a time.

## References

[1] Botker HE, Kharbanda R, Schmidt MR, Bottcher M, Kaltoft AK, Terkelsen CJ, Munk K, Andersen NH, Hansen TM, Trautner S, Lassen JF, Christiansen EH, Krusell LR, Kristensen SD, Thuesen L, Nielsen SS, Rehling M, Sorensen HT, Redington AN, Nielsen TT (2010) Remote ischaemic conditioning before hospital admission, as a complement to angioplasty, and effect on myocardial salvage in patients with acute myocardial infarction: a randomised trial. Lancet 375:727–734. 10.1016/S0140-6736(09)62001-8 20189026 10.1016/S0140-6736(09)62001-8

[2] Buehning F, Lerchner T, Vogel J, Hendgen-Cotta UB, Totzeck M, Rassaf T, Michel L (2025) Preclinical models of cardiotoxicity from immune checkpoint inhibitor therapy. Basic Res Cardiol 120:171–185. 10.1007/s00395-024-01070-0 39039301 10.1007/s00395-024-01070-0 PMC11790694

[3] Diaz-Guerra A, Clemente-Moragon A, Pollan A, Lopez-Palomar L, Cadiz L, Ibanez B (2026) Remote ischemic conditioning protects against anthracycline cardiotoxicity without impairing its antitumor activity. Basic Res Cardiol. 10.1007/s00395-026-01160-1 10.1007/s00395-026-01160-1 41686252

[4] Ferdinandy P, Andreadou I, Baxter GF, Botker HE, Davidson SM, Dobrev D, Gersh BJ, Heusch G, Lecour S, Ruiz-Meana M, Zuurbier CJ, Hausenloy DJ, Schulz R (2023) Interaction of cardiovascular nonmodifiable risk factors, comorbidities and comedications with ischemia/reperfusion injury and cardioprotection by pharmacological treatments and ischemic conditioning. Pharmacol Rev 75:159–216. 10.1124/pharmrev.121.000348 36753049 10.1124/pharmrev.121.000348 PMC9832381

[5] Galan-Arriola C, Villena-Gutierrez R, Higuero-Verdejo MI, Diaz-Rengifo IA, Pizarro G, Lopez GJ, Molina-Iracheta A, Perez-Martinez C, Garcia RD, Gonzalez-Calle D, Lobo M, Sanchez PL, Oliver E, Cordoba R, Fuster V, Sanchez-Gonzalez J, Ibanez B (2021) Remote ischaemic preconditioning ameliorates anthracycline-induced cardiotoxicity and preserves mitochondrial integrity. Cardiovasc Res 117:1132–1143. 10.1093/cvr/cvaa181 32597960 10.1093/cvr/cvaa181 PMC7983009

[6] Gaspar A, Lourenco AP, Pereira MA, Azevedo P, Roncon-Albuquerque R Jr., Marques J, Leite-Moreira AF (2018) Randomized controlled trial of remote ischaemic conditioning in ST-elevation myocardial infarction as adjuvant to primary angioplasty (RIC-STEMI). Basic Res Cardiol 113:14. 10.1007/s00395-018-0672-3 29516192 10.1007/s00395-018-0672-3

[7] Hauerslev M, Mork SR, Pryds K, Contractor H, Hansen J, Jespersen NR, Johnsen J, Heusch G, Kleinbongard P, Kharbanda R, Botker HE, Schmidt MR (2018) Influence of long-term treatment with glyceryl trinitrate on remote ischemic conditioning. Am J Physiol Heart Circ Physiol 315:H150–H158. 10.1152/ajpheart.00114.2018 29569958 10.1152/ajpheart.00114.2018

[8] Hausenloy DJ, Chilian W, Crea F, Davidson SM, Ferdinandy P, Garcia-Dorado D, van Royen N, Schulz R, Heusch G (2019) The coronary circulation in acute myocardial ischaemia/reperfusion injury: a target for cardioprotection. Cardiovasc Res 115:1143–1155. 10.1093/cvr/cvy286 30428011 10.1093/cvr/cvy286 PMC6529918

[9] Hausenloy DJ, Garcia-Dorado D, Botker HE, Davidson SM, Downey J, Engel FB, Jennings R, Lecour S, Leor J, Madonna R, Ovize M, Perrino C, Prunier F, Schulz R, Sluijter JPG, Van Laake LW, Vinten-Johansen J, Yellon DM, Ytrehus K, Heusch G, Ferdinandy P (2017) Novel targets and future strategies for acute cardioprotection: position paper of the European Society of Cardiology Working Group on Cellular Biology of the Heart. Cardiovasc Res 113:564–585. 10.1093/cvr/cvx049 28453734 10.1093/cvr/cvx049

[10] Hausenloy DJ, Kharbanda RK, Moller UK, Ramlall M, Aaroe J, Butler R, Bulluck H, Clayton T, Dana A, Dodd M, Engstrom T, Evans R, Lassen JF, Christensen EF, Garcia-Ruiz JM, Gorog DA, Hjort J, Houghton RF, Ibanez B, Knight R, Lippert FK, Lonborg JT, Maeng M, Milasinovic D, More R, Nicholas JM, Jensen LO, Perkins A, Radovanovic N, Rakhit RD, Ravkilde J, Ryding AD, Schmidt MR, Riddervold IS, Sorensen HT, Stankovic G, Varma M, Webb I, Terkelsen CJ, Greenwood JP, Yellon DM, Botker HE, Investigators C-E-P (2019) Effect of remote ischaemic conditioning on clinical outcomes in patients with acute myocardial infarction (CONDI-2/ERIC-PPCI): a single-blind randomised controlled trial. Lancet 394:1415–1424. 10.1016/S0140-6736(19)32039-2 31500849 10.1016/S0140-6736(19)32039-2 PMC6891239

[11] Hausenloy DJ, Yellon DM (2016) Ischaemic conditioning and reperfusion injury. Nat Rev Cardiol 13:193–209. 10.1038/nrcardio.2016.5 26843289 10.1038/nrcardio.2016.5

[12] Heusch G (2018) 25 years of remote ischemic conditioning: from laboratory curiosity to clinical outcome. Basic Res Cardiol 113:15. 10.1007/s00395-018-0673-2 29516255 10.1007/s00395-018-0673-2

[13] Heusch G (2019) Coronary microvascular obstruction: the new frontier in cardioprotection. Basic Res Cardiol 114:45. 10.1007/s00395-019-0756-8 31617010 10.1007/s00395-019-0756-8

[14] Heusch G (2017) Critical issues for the translation of cardioprotection. Circ Res 120:1477–1486. 10.1161/CIRCRESAHA.117.310820 28450365 10.1161/CIRCRESAHA.117.310820

[15] Heusch G (2020) Myocardial ischaemia-reperfusion injury and cardioprotection in perspective. Nat Rev Cardiol 17:773–789. 10.1038/s41569-020-0403-y 32620851 10.1038/s41569-020-0403-y

[16] Heusch G, Andreadou I, Bell R, Bertero E, Botker HE, Davidson SM, Downey J, Eaton P, Ferdinandy P, Gersh BJ, Giacca M, Hausenloy DJ, Ibanez B, Krieg T, Maack C, Schulz R, Sellke F, Shah AM, Thiele H, Yellon DM, Di Lisa F (2023) Health position paper and redox perspectives on reactive oxygen species as signals and targets of cardioprotection. Redox Biol 67:102894. 10.1016/j.redox.2023.102894 37839355 10.1016/j.redox.2023.102894 PMC10590874

[17] Heusch G, Botker HE, Przyklenk K, Redington A, Yellon D (2015) Remote ischemic conditioning. J Am Coll Cardiol 65:177–195. 10.1016/j.jacc.2014.10.031 25593060 10.1016/j.jacc.2014.10.031 PMC4297315

[18] Heusch G, Gersh BJ (2016) ERICCA and RIPHeart: two nails in the coffin for cardioprotection by remote ischemic conditioning? Probably not! Eur Heart J 37:200–202. 10.1093/eurheartj/ehv606 26508160 10.1093/eurheartj/ehv606

[19] Heusch G, Gersh BJ (2020) Is cardioprotection salvageable? Circulation 141:415–417. 10.1161/CIRCULATIONAHA.119.044176 32078426 10.1161/CIRCULATIONAHA.119.044176

[20] Heusch G, Kleinbongard P (2025) Ischaemic conditioning: a renaissance with wider perspectives? Eur Heart J 46:2076–2078. 10.1093/eurheartj/ehaf205 40197755 10.1093/eurheartj/ehaf205

[21] Heusch G, Kleinbongard P, Rassaf T (2019) Cardioprotection beyond infarct size reduction. Circ Res 124:679–680. 10.1161/CIRCRESAHA.119.314679 30817254 10.1161/CIRCRESAHA.119.314679

[22] Heusch G, Rassaf T (2021) Protection from cardiotoxicity of cancer chemotherapy: a novel target for remote ischaemic conditioning? Cardiovasc Res 117:985–986. 10.1093/cvr/cvaa199 32637985 10.1093/cvr/cvaa199

[23] Heusch G, Rassaf T (2016) Time to give up on cardioprotection? A critical appraisal of clinical studies on ischemic pre-, post-, and remote conditioning. Circ Res 119:676–695. 10.1161/CIRCRESAHA.116.308736 27539973 10.1161/CIRCRESAHA.116.308736

[24] Kleinbongard P, Botker HE, Ovize M, Hausenloy DJ, Heusch G (2020) Co-morbidities and co-medications as confounders of cardioprotection-does it matter in the clinical setting? Br J Pharmacol 177:5252–5269. 10.1111/bph.14839 31430831 10.1111/bph.14839 PMC7680006

[25] Korste S, Settelmeier S, Michel L, Odersky A, Stock P, Reyes F, Haj-Yehia E, Anker MS, Gruneboom A, Hendgen-Cotta UB, Rassaf T, Totzeck M (2023) Anthracycline therapy modifies immune checkpoint signaling in the heart. Int J Mol Sci. 10.3390/ijms24076052 37047026 10.3390/ijms24076052 PMC10094326

[26] Mallouppas M, Chung R, Ghosh AK, Macklin A, Yellon DM, Walker JM (2023) Anthracyclines and biomarkers of myocardial injury: the effect of remote ischemic conditioning. JACC CardioOncol 5:343–355. 10.1016/j.jaccao.2023.03.008 37397080 10.1016/j.jaccao.2023.03.008 PMC10308041

[27] Meybohm P, Bein B, Brosteanu O, Cremer J, Gruenewald M, Stoppe C, Coburn M, Schaelte G, Boning A, Niemann B, Roesner J, Kletzin F, Strouhal U, Reyher C, Laufenberg-Feldmann R, Ferner M, Brandes IF, Bauer M, Stehr SN, Kortgen A, Wittmann M, Baumgarten G, Meyer-Treschan T, Kienbaum P, Heringlake M, Schon J, Sander M, Treskatsch S, Smul T, Wolwender E, Schilling T, Fuernau G, Hasenclever D, Zacharowski K, Collaborators RIS (2015) A multicenter trial of remote ischemic preconditioning for heart surgery. N Engl J Med 373:1397–1407. 10.1056/NEJMoa1413579 26436208 10.1056/NEJMoa1413579

[28] Rassaf T, Anker SD, Anker MS (2025) The convergence of two epidemics - unravelling the diagnostic complexities at the heart of cardio-oncology. Eur J Heart Fail 27:2112–2115. 10.1002/ejhf.3774 40808518 10.1002/ejhf.3774

[29] Rassaf T, Totzeck M, Backs J, Bokemeyer C, Hallek M, Hilfiker-Kleiner D, Hochhaus A, Luftner D, Muller OJ, Neudorf U, Pfister R, von Haehling S, Lehmann LH, Bauersachs J, Committee for Clinical Cardiovascular Medicine of the German Cardiac S (2020) Onco-cardiology: consensus paper of the German cardiac society, the German society for pediatric cardiology and congenital heart defects and the German society for hematology and medical oncology. Clin Res Cardiol 109:1197–1222. 10.1007/s00392-020-01636-7 32405737 10.1007/s00392-020-01636-7 PMC7515958

[30] Rassaf T, Totzeck M, Hendgen-Cotta UB, Shiva S, Heusch G, Kelm M (2014) Circulating nitrite contributes to cardioprotection by remote ischemic preconditioning. Circ Res 114:1601–1610. 10.1161/CIRCRESAHA.114.303822 24643960 10.1161/CIRCRESAHA.114.303822

[31] Sloth AD, Schmidt MR, Munk K, Kharbanda RK, Redington AN, Schmidt M, Pedersen L, Sorensen HT, Botker HE, Investigators C (2014) Improved long-term clinical outcomes in patients with ST-elevation myocardial infarction undergoing remote ischaemic conditioning as an adjunct to primary percutaneous coronary intervention. Eur Heart J 35:168–175. 10.1093/eurheartj/eht369 24031025 10.1093/eurheartj/eht369

[32] Totzeck M, Hendgen-Cotta UB, French BA, Rassaf T (2016) A practical approach to remote ischemic preconditioning and ischemic preconditioning against myocardial ischemia/reperfusion injury. J Biol Methods. 10.14440/jbm.2016.149 28066791 10.14440/jbm.2016.149 PMC5218813

[33] Totzeck M, Mincu RI, Heusch G, Rassaf T (2019) Heart failure from cancer therapy: can we prevent it? ESC Heart Fail 6:856–862. 10.1002/ehf2.12493 31297946 10.1002/ehf2.12493 PMC6676296

[34] Totzeck M, Schuler M, Stuschke M, Heusch G, Rassaf T (2019) Cardio-oncology - strategies for management of cancer-therapy related cardiovascular disease. Int J Cardiol 280:163–175. 10.1016/j.ijcard.2019.01.038 30661849 10.1016/j.ijcard.2019.01.038

[35] Yeh ET, Tong AT, Lenihan DJ, Yusuf SW, Swafford J, Champion C, Durand JB, Gibbs H, Zafarmand AA, Ewer MS (2004) Cardiovascular complications of cancer therapy: diagnosis, pathogenesis, and management. Circulation 109:3122–3131. 10.1161/01.CIR.0000133187.74800.B9 15226229 10.1161/01.CIR.0000133187.74800.B9

